# Editorial: Circuit Mechanisms of Neurodegenerative Diseases

**DOI:** 10.3389/fnins.2020.593329

**Published:** 2020-09-30

**Authors:** Smita Saxena, Sabine Liebscher

**Affiliations:** ^1^Department of Neurology, Center for Experimental Neurology, Bern University Hospital, Bern, Switzerland; ^2^Institute of Clinical Neuroimmunology, Klinikum der Universität München, Ludwig-Maximilians University Munich, Martinsried, Germany; ^3^Munich Cluster for Systems Neurology (SyNergy), Munich, Germany; ^4^Biomedical Center, Ludwig-Maximilians University Munich, Martinsried, Germany

**Keywords:** neurodegeneration, neural circuits, Parkinson's disease, Huntington's disease, amyotrophic lateral sclerosis (ALS), spinocerebellar ataxia (SCA), interneurons, Alzheimer's disease

Neurodegenerative diseases (NDs), such as Alzheimer's disease (AD), Parkinson's disease (PD), Huntington's disease (HD), or Amyotrophic lateral sclerosis (ALS) are histopathologically characterized by the formation of protein aggregates and the selective death of a defined population of neurons. These diseases have traditionally been viewed as being caused by the selective dysfunction of those vulnerable neurons via cell autonomous mechanisms. Current evidence, however, strongly implicates a causal involvement of altered neuronal circuit function in triggering and perpetuating the degenerative cascade. Recent methodological advances offered by newly developed genetic tools, *in vivo* electrophysiology and imaging techniques, enabled the selective assessment and manipulation of dedicated cell populations and neural circuits *in vivo*. Such approaches have provided evidence that circuit alterations result from complex changes at the level of synapses, intrinsic excitability of cells and disrupted connectivity within local microcircuits and between projection areas. This ultimately results in altered activity patterns of neurons, impaired information processing in a disease-stage dependent manner and eventual neuronal loss. Intriguingly, preclinical studies in rodent models suggest that many of those structural and functional alterations within a neuronal network are already found at early presymptomatic stages, long before typical markers of degeneration are detectable. These findings support the notion that circuit dysfunction is likely not only a consequence of degenerative processes in NDs, but can in fact represent a main driver of the pathology. This novel concept not only opens up new therapeutic avenues by shedding light into brain-region, and cell-type specific alterations occurring in a disease-stage specific manner; but moreover, it has the potential to identify novel diagnostic approaches and therapeutic windows.

In this Research Topic Werner et al. summarize the current status and opportunities of *in vivo* imaging using miniature fluorescence microscopes (miniscopes), which enables chronic monitoring of neuronal activity and the disentangling of circuit deficits in NDs. A number of review articles summarize the current knowledge about the impact of neurodegenerative processes on neural circuit function and development. As such, Blumenstock and Dudanova explore cell-type specific impairments in cortical and striatal circuits in HD, while Binda et al. summarize evidence arguing for developmental deficits in the maturation of cerebellar circuits in Spinocerebellar Ataxias. Gunes et al. recapitulate findings of altered excitability in various elements of motor circuits in ALS and Brunet et al. assemble evidence for cortical dysfunction as a main driver of ALS pathophysiology. Another set of papers addresses the circuit mechanism of memory loss in mouse models of dementia. To this end, Vyas et al. review the complex structural and functional changes of single neurons and neural circuits in hippocampus of mouse models of AD. Yeung et al. investigate the impact of amyloid-beta 42, one of the main molecular players in AD, on glutamatergic receptor and transporter expression in hippocampus. In a systematic review Rashidi-Ranjbar et al. revisit evidence for structural and functional impairments of corticolimbic circuits in late-life depression and dementia. Furthermore, in a hypothesis paper Janitzky argues for a compromised function of the locus coereleus (LC)—a brain region harboring noradrenergic projection neurons—in early stages of NDs. LC dysfunction results in persistent high tonic discharge, while the phasic discharge is impaired, which is hypothesized to diminish putative anti-inflammatory and neuroprotective effects conveyed by phasic LC firing.

With this Research Topic in Frontiers in Neuroscience, we aim to provide compelling recent evidence in support of the hypothesis of altered circuit function as a potent co-driver of various NDs. This volume contains original research articles as well as reviews providing an overview of the current knowledge and technological advances in the mentioned area of neurodegeneration.

## Author Contributions

SS and SL were equally involved in the conceptualization and editing of the Research Topic.

## Conflict of Interest

The authors declare that the research was conducted in the absence of any commercial or financial relationships that could be construed as a potential conflict of interest.

